# Clinical Features of the Aslanger Pattern to Compensate for the Limitation of ST-Elevation Myocardial Infarction (STEMI) Criteria

**DOI:** 10.7759/cureus.33227

**Published:** 2023-01-01

**Authors:** Eiji Miyauchi, Kota Kuwazuru, Ryo Arikawa, Daisuke Tokutake, Hideto Chaen, Naoya Oketani, Mitsuru Ohishi

**Affiliations:** 1 Cardiology, Kagoshima City Hospital, Kagoshima, JPN; 2 Cardiovascular Medicine and Hypertension, Graduate School of Medical and Dental Sciences, Kagoshima, JPN

**Keywords:** door-to-catheterization laboratory time, door-to-balloon time, acs ( acute coronary syndrome ), non-st-elevation myocardial infarction (nstemi), aslanger pattern

## Abstract

Background: ST-elevation is one of the most valuable electrocardiogram findings to diagnose acute myocardial infarction. However, more than a quarter of acute coronary occlusions are missed by this criterion, causing a delay in revascularization. Therefore, there should be awareness of the limitations of the current criteria and new electrocardiographic findings are required as a diagnostic tool to compensate for them. The Aslanger pattern is a specific electrocardiographic finding in acute inferior myocardial infarction with multivessel disease and allows the detection of inferior myocardial infarction that does not show ST-elevation, leading to rapid revascularization. However, in patients with the Aslanger pattern, the hemodynamic characteristics, such as the rate of shock and the use of mechanical circulatory support, as well as prognostic characteristics such as the in-hospital mortality rate, have not yet been clarified.

Methods: In this study, we retrospectively surveyed the current practice on the basis of ST-elevation myocardial infarction (STEMI) criteria in patients with acute coronary artery occlusion presenting with inferior myocardial infarction. We examined the clinical characteristics of the Aslanger pattern.

Results: Based on the STEMI criteria, 71.8% (51/72) of patients were diagnosed with STEMI from an acute electrocardiogram, and 28.2% (21/78) were diagnosed with non-STEMI. As expected, ruling out in all acute coronary artery occlusions using STEMI criteria alone was difficult. A total of 48% of patients with non-STEMI had the Aslanger pattern. In addition, 80% of patients with the Aslanger pattern had multivessel disease, 30% had the use of the mechanical circulatory support, and 20% had in-hospital mortality.

Conclusion: This study suggests that the Aslanger pattern is useful not only for diagnosis, but also for predicting hemodynamic collapse and a poor prognosis. Therefore, we should share information on Aslanger pattern with other physicians and use this pattern in daily practice.

## Introduction

The ST-elevation myocardial infarction (STEMI) criteria (4th universal definition reported by Thygesen et al. [1]) have become the established criteria for STEMI. However, Khan et al. reported that more than one quarter of acute coronary artery occlusions are missed by these criteria, causing a delay in revascularization [2]. Therefore, there needs to be awareness of the limitations of the current criteria. Additionally, new electrocardiographic findings are required as a diagnostic method to compensate for these limitations.

Tahvanainen et al. reported that one of the factors causing limitations of the current STEMI criteria is a multivessel disease [3]. In cases of multivessel disease, ST-segment elevation caused by occluded vessels may be difficult to recognize because the ST segment is also depressed by other ischemic stenotic vessels causing ST-segment depression. The ST-segment elevation and depression may cancel each other out. As a result, despite the severity of the disease, the revascularization may be delayed owing to the indistinct ST-T findings.

Aslanger et al. reported a new electrocardiographic finding in 2020 [4]. This pattern is a specific electrocardiographic finding in acute inferior myocardial infarction with multivessel disease and allows the detection of inferior myocardial infarction that does not meet STEMI criteria, leading to rapid revascularization. The Aslanger pattern is an innovative diagnostic tool, which focuses on subtle electrocardiogram (ECG) changes only using general 12-lead recordings, without using special recording methods such as right-sided leads. Patients with the Aslanger pattern often have acute inferior myocardial infarction with multivessel disease. Therefore, many of these patients are likely to have hemodynamic deterioration and require induction of mechanical circulatory support. However, the clinical features of the patients with the Aslanger pattern have not yet been reported.

In this study, we retrospectively surveyed the current practice using STEMI criteria in patients with acute coronary artery occlusion who presented with inferior myocardial infarction and examined the clinical characteristics of the Aslanger pattern.

## Materials and methods

We retrospectively examined the proportion of patients with the Aslanger pattern in non-STEMI (NSTEMI) and the clinical characteristics of patients with the Aslanger pattern in adults aged 18 years or older who were diagnosed with acute inferior myocardial infarction. This study included patients at our hospital between 1 January 2020 and 31 June 2022. Patients whose coronary lesions were not evaluated by coronary angiography and/or who had a history of coronary artery bypass grafting were excluded.

All clinical information was collected from the medical records. Every ECG was reviewed by two cardiologists who were blinded to the angiographic and clinical outcomes. In case of discrepancies in findings, a third cardiologist read and adjusted the reading. The ST elevation was defined according to the 4th universal definition. The Aslanger pattern was defined as (1) any ST elevation in lead III, but not in other inferior leads, (2) ST depression in any of the leads V4 to V6 (but not in V2) with a positive or terminally positive T-wave, and (3) ST in lead V1 is higher than ST in V2. Coronary angiograms were reviewed by two cardiologists who were blinded to the ECGs. In case of discrepancies in findings, a third cardiologist read and adjusted the reading. The culprit coronary artery was defined as a vessel with acute thrombotic total or subtotal occlusion. With regard to stenotic lesions, 75% or more stenosis was considered severe stenosis. The hospital arrival time (door time), catheterization laboratory arrival time (catheterization laboratory time), and device insertion time (balloon time) were collected. The door-to-catheterization laboratory time (DTCT), catheterization laboratory-to-balloon time (CTBT), and door-to-balloon time (DTBT) were defined as the time from hospital arrival to device insertion.

The requirement for informed consent was waived owing to the anonymous nature of the data. Approval for the study was obtained from our Institutional Review Board (approval number 2022-20).

Continuous, normally distributed data are expressed as the means ± standard deviation, and skewed data as the medians and the first and third quartiles. Categorical data are shown as the number (percentage). The Student’s t test and Wilcoxon rank-sum test were used for normally and non-normally distributed continuous data, respectively. Categorical variables were compared using the chi-square test or Fisher’s exact test as appropriate. In the analysis of factors affecting the DTBT, potentially confounding factors were selected from clinical findings and previous studies. All statistical analyses were performed with JMP® Pro version 11 (SAS Institute, Inc., Cary, NC, USA) for Windows.

## Results

Seventy-nine patients were diagnosed with acute inferior myocardial infarction between 1 January 2020 and 31 June 2022, at our hospital. Seventy-two patients were included in the analysis, after excluding seven patients who did not undergo coronary catheterization and treatment in the acute phase. Fifty-one (71.8%) patients had STEMI and 20 (29.2%) patients had NSTEMI (Table 1).

**Table 1:** Baseline patient characteristics. STEMI, ST elevation myocardial infarction; NSTEMI, non-STEMI; PCI, percutaneous coronary intervention

	STEMI (n=51)	NSTEMI (n=20)	p value
Age (years)	70 (41-94)	71 (51-97)	0.49
Gender (male) [n(%)]	35 (68.6%)	18 (90.0%)	0.07
Body mass index (kg/m2)	23.7 (14.0-41.7)	23.0 (16.5-31.4)	0.47
Hypertension [n(%)]	32 (62.7%)	17 (85.0%)	0.09
Dyslipidemia [n(%)]	22 (43.1%)	10 (50.0%)	0.80
Diabetes mellitus [n(%)]	12 (23.5%)	9 (45.0%)	0.15
Current smoke [n(%)]	7 (13.7%)	2 (10.0%)	1.00
Chronic kidney disease [n(%)]	10 (19.6%)	3 (15.0%)	0.74
Hemodialysis [n(%)]	0 (0.0%)	0 (0.0%)	NA
Prior PCI [n(%)]	1 (2.0%)	3 (15.0%)	0.02

The overall median DTBT was 86 min (73-118 min) (Table 2).

**Table 2:** DTBT and two components of DTBT. STEMI, ST elevation myocardial infarction; NSTEMI, non-ST elevation myocardial infarction; DTBT, door-to-balloon-time; DTCT, door-to-catheter laboratory-time; CTBT, catheter laboratory-to-balloon-time

	Overall (n=72)	STEMI (n=51)	NSTEMI (n=20)	p value
DTBT (min)	86 (73-118)			
		83 (71-104)	137 (87-201)	<0.01
DTCT (min)	45 (37-65)			
		35 (41-50)	72 (52-172)	<0.01
CTBT (min)	39 (34-52)			
		40 (36-51)	39 (32-66)	0.91
DTBT<90 [n(%)]	39 (54.1%)			
		33 (64.7%)	6 (28.5%)	0.04

The median DTBT was significantly lower for STEMI than NSTEMI (p<0.01). When the DTBT was divided into two components, the DTCT and the CTBT, we found that the median DTCT was significantly lower for STEMI than NSTEMI (p<0.01). The median CTBT for STEMI was not significantly different to that for NSTEMI. The DTBT within 90 min (DTBT<90) was significantly higher for STEMI than NSTEMI (p=0.04). We determined that the DTCT was the primary determinant of the DTBT and examined factors affecting the DTCT. In a univariate analysis, “obtaining a prehospital ECG” and “meeting STEMI criteria” were associated with a shorter DTCT (Table 3) (p=0.02, p<0.01, respectively).

**Table 3:** Univariate analysis. PCI, percutaneous coronary intervention; ECG, electrocardiography; STEMI, ST elevation myocardial infarction; ED, emergency department; CT, computed tomography

Variable	Estimates	95% CI	p value
Out-of-hour visit	23.1	-10.2–56.5	0.17
Doctor experience > 5 years	-19.5	-72.3–33.3	0.46
Prior PCI	94.6	33.0–156.1	<0.01
Typical ischemic symptom	-19.7	-67.7–28.2	0.41
Prehospital ECG	-39.1	-72.8–-5.3	0.02
Meets STEMI criteria	-74.8	-106.9–-42.8	<0.01
Cardiac arrest at ED	-32.2	-115.2–50.8	0.44
Shock at ED	-19.1	-62.2–24.0	0.37
Intubated at ED	-9.6	-92.6–73.7	0.81
CT at ED	41.2	-23.5–106.0	0.20

In addition, “prior primary coronary intervention” was associated with a prolonged DTCT. A multivariate analysis of potential confounders showed that “meeting STEMI criteria” was associated with shortening of the DTCT (p<0.01), but not with prolongation of the DTCT (Table 4).

**Table 4:** Multivariate analysis. PCI, percutaneous coronary intervention; ECG, electrocardiography; STEMI, ST elevation myocardial infarction

Variable	Estimates	95% CI	p value
Prior PCI	23.9	-6.8–54.7	0.13
Prehospital ECG	49.1	-11.1–109.2	0.11
Meets STEMI criteria	-62.4	-95.5–-19.4	<0.01

Among the factors independently associated with a change in the DTCT, only “meeting STEMI criteria” was associated with a significant change in the DTCT (p<0.01). In patients with NSTEMI, 50% (10/20) had the Aslanger pattern, and 50% (10/20) did not show the Aslanger pattern (Table 5).

**Table 5:** NSTEMI with Aslanger pattern vs NSTEMI without Aslanger pattern. NSTEMI, non-ST elevation myocardial infarction; ED, emergency department;  DTCT, door-to-catheter laboratory-time; DTBT, door-to-balloon-time; RCA, right coronary artery; LCX, left circumflex artery; LAD, left anterior descending artery; LMT, left main trunk; TIMI flow, thrombolysis in myocardial infarction flow; CL, catheter laboratory; MCS, mechanical circulatory support; IABP, intra aortic balloon pumping; PCPS, percutaneous cardiopulmonary support

		NSTEMI (n=20)		
		with Aslanger pattern (n=10)	without Aslanger pattern (n=10)	p value
Troponin at ED				
TpI (pg/mL)		260.2 (99.0-2009.3)	2320.8 (432.5-4177.6)	0.35
TpT positive [n(%)]		3 (30.0%)	5 (50.0%)	0.62
Time				
DTCT (min)		69 (43.2-88.8)	76 (58.5-186.5)	0.43
DTBT (min)		148.5 (78.5-187)	115.0 (91.0-205)	0.60
DTBT<90 [n(%)]		4 (40.0%)	2 (20.0%)	0.36
Culprit artery				1.0
RCA [n(%)]		8 (80.0%)	8 (80.0%)	
LCX [n(%)]		2 (20.0%)	3 (30.0%)	
LAD [n(%)]		0 (0.0%)	0 (0.0%)	
LMT [n(%)]		0 (0.0%)	0 (0.0%)	
Number of diseased vessel				0.30
One vessel [n(%)]		2 (20.0%)	4 (40.0%)	
Two vessels [n(%)]		1 (10.0%)	3 (30.0%)	
Three vessels [n(%)]		7 (70.0%)	4 (40.0%)	
Initial TIMI flow				1.0
0 [n(%)]		5 (50.0%)	5 (50.0%)	
1 [n(%)]		5 (50.0%)	6 (60.0%)	
>2 [n(%)]		0 (0.0%)	0 (0.0%)	
Hemodynamics				
Shock at ED [n(%)]		1 (10.0%)	0 (0.0%)	0.48
Shock at CL [n(%)]		4 (40.0%)	2 (20.0%)	0.36
MCS				
IABP [n(%)]		3 (30.0%)	0 (0.0%)	0.09
PCPS [n(%)]		1 (10.0%)	0 (0.0%)	0.48
Prognosis				
In-hospital mortality [n(%)]		2 (20.0%)	0(0.0%)	0.21

The positive rate of troponin (TpT) was 30% (3/10) in patients with the Aslanger pattern and 50.0% (5/10) in those without the Aslanger pattern (p=0.62). The absolute value of troponin I (TpI) was 260.2 pg/mL in patients with the Aslanger pattern and 2320.8 pg/mL in those without the Aslanger pattern (p=0.35). Culprit lesions were predominantly right coronary arteries with or without the Aslanger pattern, and the thrombolysis in myocardial infarction flow grades were not significantly different between patients with and without the Aslanger pattern (p=1.0).

In patients with NSTEMI and the Aslanger pattern, 10% (1/10) had two-vessel lesions, 70% (7/10) had three-vessel lesions, and 80% (8/10) had multivessel lesions (Table 5). However, in patients with NSTEMI without the Aslanger pattern, 30.0% (3/10) had two-vessel lesions, 40.0% (4/10) had three-vessel lesions, and 70.0% (7/10) had multivessel lesions. The proportion of patients with multiple lesions tended to be higher in patients with the Aslanger pattern than in those without the Aslanger pattern (p=0.30). Cardiogenic shock at the Emergency Department was present in 10% (1/10) of patients with the Aslanger pattern, but not in those without the Aslanger pattern (p=0.48). Cardiogenic shock in the catheterization laboratory was observed in 40% (4/10) of patients with the Aslanger pattern and in 20.0% (2/10) of patients without the Aslanger pattern (p=0.36). In both situations, cardiogenic shock tended to occur in patients presenting with the Aslanger pattern. Intra-aortic balloon pumping was used in 30% (3/10) of patients with the Aslanger pattern and in 0% (0/10) of patients without the Aslanger pattern (p=0.09). Percutaneous cardiopulmonary support use was 10% (1/10) in patients with the Aslanger pattern and 0% (0/10) in those without the Aslanger pattern (p=0.48). The use of all mechanical circulatory support tended to be higher in patients with the Aslanger pattern than in those without the Aslanger pattern. The in-hospital mortality rate was 20% (2/10) in patients with the Aslanger pattern and 0% (0/10) in those without the Aslanger pattern (p=0.21). The short-term prognosis tended to be higher in patients with the Aslanger pattern than in those without the Aslanger pattern.

## Discussion

In this study, the factor that strongly affected the DTCT was “meeting STEMI criteria.” This finding suggests that STEMI criteria are a diagnostic tool in the decision-making process in the treatment of acute coronary syndrome as previously reported by O’Gara et al. [5].

However, while all patients in this study had acute coronary artery occlusion, 28.2% (20/71) were diagnosed with NSTEMI on the basis of STEMI criteria. STEMI criteria alone may not be sufficient to rule in all acute coronary artery occlusions. Aslanger et al. reported that we needed to recognize subtle ECG changes in treating in the patients with acute coronary syndrome [6-9]. Therefore, Pendell Meyers et al. reported that other diagnostic tools need to be determined to compensate for STEMI criteria [10]. TpT is a useful tool for diagnosing acute coronary syndrome and providing the results in approximately 15 min, but its sensitivity is relatively low, especially in the immediately after the onset of acute coronary syndrome reported by Kontos et al. [11] and Smilowitz et al. [12]. In this study, 65.0% (13/20) of patients with NSTEMI were TpT negative and had to wait for TpI testing.

The Aslanger pattern is an electrocardiographic finding of ST changes, and its usefulness is not expected to decline, even in the early stages of the onset of myocardial infarction [4]. Therefore, the Aslanger pattern may have clinical significance as a clinical test that is different from TpT. In this study, only 30% (3/10) of patients with the Aslanger pattern had a positive result for TpT. If a patient shows the Aslanger pattern, the indication for urgent revascularization may be determined without waiting for testing of TpT, regardless of its result. Therefore, the Aslanger pattern complements STEMI criteria and may contribute to improving the diagnostic yield of acute coronary artery occlusion.

Among the patients with the Aslanger pattern in this study, 80% had multivessel disease (STEMI: 41%, NSTEMI without the Aslanger pattern: 63%). As expected, the presence of multiple vessel disease may complicate the ECG findings and lead to ECGs that do not meet STEMI criteria.

Zhelev et al. reported that multivessel vessel disease might have an effect on the hemodynamic status [13]. Günlü and Demir reported that in case to treat with fibrinolytic agents, administration of tenecteplase in STEMI patients who received a loading dose with ticagrelor resulted in a significant reduction in major adverse cardiac events compared to alteplase [14]. In this study, patients with the NSTEMI and the Aslanger pattern tended to have a relatively high incidence of hemodynamic deterioration during revascularization procedures compared with those without the Aslanger pattern. The use of all mechanical circulatory support tended to be higher in patients with NSTEMI amd the Aslanger pattern than in those without the Aslanger pattern. The in-hospital mortality rate of patients with NSTEMI and the Aslanger pattern was 20.0% (2/10) and the causes of death were cardiogenic shock. These patients may have been unable to maintain their hemodynamic status owing to extensive necrosis of the myocardium in multivessel lesions. 

Our findings suggest that the Aslanger pattern is useful not only as a diagnostic tool to complement STEMI criteria, but also as a predictive and prognostic tool for a worsening hemodynamic status.

There are several limitations to this study. The number of patients in this study was small. Further studies with a larger number of patients are requires to better understand the characteristics of the Aslanger pattern more accurately. The level of acute care differs according to the size of the facility, which may affect the variation in the hemodynamic status. Prospective studies are warranted to evaluate the clinical value of the STEMI criteria with the Aslanger pattern.

## Conclusions

This study suggests that the Aslanger pattern may be useful not only as a diagnostic tool to complement STEMI criteria, but also for predicting the hemodynamic status and the acute prognosis. Patients with the Aslanger pattern have acute inferior myocardial infarction with multivessel disease, and many of these patients have hemodynamic deterioration and require induction of mechanical circulatory support. Therefore, sharing information on the Aslanger pattern with other physicians and using this pattern might be of great clinical significance.
